# Supplementary material for: Quality Formation of Adzuki Bean Baked: From Acrylamide to Volatiles under Microwave Heating and Drum Roasting
Source: Foods. 2021 Nov 10;10(11):2762. doi: 10.3390/foods10112762 (PMC8621577; doi:10.3390/foods10112762)
Supplement: Supplementary file 1 [file foods-10-02762-s001.zip › foods-1402175-supplementary.pdf]

Supplementary Materials

Table S1. Identified volatile compounds under microwave baking and drum roasting

Category	Control	Microwave baking						Drum roasting								
		2 min	4 min	6 min	8 min	10 min	12 min	6 min 109.6 ℃	7 min 120.3 ℃	8 min 124.6 ℃	10 min 135.1 ℃	12 min 141.3 ℃	13 min 144.8 ℃	15 min 150.7 ℃	17 min 155.0 ℃	23 min 158.9 ℃
**Aldehyde (mg/kg)**	0.4469±0.0389	0.8003±0.0696	0.6145±0.0381	0.3320±0.0272	1.1427±0.1257	0.1421±0.0068	0.5015±0.0286	0.7551±0.0680	1.0504±0.0336	0.7028±0.0323	0.5290±0.0291	2.2807±0.0411	2.6157±0.0497	1.7635±0.0370	0.7600±0.0327	0.1214±0.0063
**Alcohol (mg/kg)**	0.1669±0.0114	0.0470±0.0032	0.4094±0.0254	0.0421±0.0035	0.3396±0.0204	0.0700±0.0034	0.1474±0.0084	0.0217±0.0017	0.5575±0.0184	0.0958±0.0073	0.2301±0.0127	0.5734±0.0183	0.3251±0.0126	0.3513±0.0126	0.0379±0.0016	0.1561±0.0081
**Pyrazine (mg/kg)**	0.0400±0.0017	0.0104±0.0007	-	-	-	0.0282±0.0014	-	0.2237±0.0174	-	-	-	0.1295±0.0097	1.5633±0.1485	2.7014±0.1864	2.3570±0.2098	2.0512±0.1579
**Ester (mg/kg)**	0.0463±0.0022	0.0372±0.0014	0.0438±0.0027	-	0.0460±0.0028	0.0553±0.0027	0.1904±0.0168	0.1562±0.0059	0.0227±0.0016	-	-	0.3925±0.0177	0.1560±0.0123	0.0410±0.0028	-	-
**Furane (mg/kg)**	-	0.3535±0.0240	0.2046±0.0127	0.1721±0.0141	0.4960±0.0298	0.0589±0.0028	0.1984±0.0113	0.3050±0.0238	0.3607±0.0260	0.3144±0.0239	0.1832±0.0101	0.6798±0.0510	0.3220±0.0254	0.3551±0.0245	0.0939±0.0040	0.0292±0.0015
